# The ProQOL-21: A revised version of the Professional Quality of Life (ProQOL) scale based on Rasch analysis

**DOI:** 10.1371/journal.pone.0193478

**Published:** 2018-02-28

**Authors:** Brody Heritage, Clare S. Rees, Desley G. Hegney

**Affiliations:** 1 School of Psychology and Exercise Science, Murdoch University, Perth, Western Australia, Australia; 2 School of Psychology, Curtin University, Perth, Western Australia, Australia; 3 Research Division, Central Queensland University, Brisbane, Queensland, Australia; 4 School of Nursing, University of Adelaide, Adelaide, South Australia, Australia; University of Antwerp, BELGIUM

## Abstract

The Professional Quality of Life scale is a measure intended to provide practitioners and researchers with an indication of a caring professional’s compassion satisfaction, burnout, and secondary traumatic stress. While this measure has been used extensively in nursing research, owing to the relevancy of patient-care associated satisfaction and fatigue within this profession, information regarding the construct validity of this measure is less well represented in the literature. We examined the construct validity of the Professional Quality of Life scale using a Rasch analysis procedure on each of its three scales, as a means of substantiating their measurement adequacy. Responses on the Professional Quality of Life scale from 1615 registered nurses (age x̅ = 46.48 years, *SD* = 11.78) were analysed. While support for the measurement adequacy (invariance, person/item fit, and unidimensionality) of the compassion satisfaction scale was found, the burnout and secondary traumatic stress scales did not demonstrate adequate measurement properties. We instead present an alternative measurement model of these subscales, involving items from each, to form a robust measure of compassion fatigue, and provide recoding, scoring, and normed scores for both measures. Our findings indicate that use of the Professional Quality of Life scale’s burnout and secondary traumatic stress scales may require caution, while our revised compassion satisfaction and fatigue scales provide robust measurement options for practitioners and researchers.

## Introduction

The Professional Quality of Life (ProQOL) [1] scale is a commonly used measure of compassion fatigue and compassion satisfaction in the nursing literature (e.g. [2]). The ProQOL is intended for use as a screening tool for the positive and negative aspects of working within a helping profession such as nursing. To this end, the ProQOL looks at two overarching factors of compassion satisfaction and compassion fatigue. Stamm [1] described compassion satisfaction as the satisfaction individuals derive from performing their work-related tasks well, in addition to satisfaction with one’s colleagues, and how one’s work has broader societal value. While focused on several aspects of satisfaction with work, and bearing similarity with constructs such as job satisfaction (e.g., "...acceptance of a (relatively) satisfactory situation"; [3]), compassion satisfaction frames the derivation of work-related satisfaction from helping or caring behaviours at work [4]. People within helping professions, such as nurses and other allied health professionals, are proposed to offset the negative aspects of their work on the basis of their compassion satisfaction [5]. While compassion satisfaction stems from interactions with patients, compassion fatigue is a less-positive alternative outcome of patient care [6]. Compassion fatigue stems from interactions with patients that leave the health professional preoccupied with the trauma experienced by the patient, and enhanced emotional arousal or avoidance behaviours associated with the patient [7]. In the conceptualisation of the ProQOL, Stamm [1] encompasses the previous behaviours and cognitions under the construct of secondary traumatic stress, suggesting that it reflects feelings of fear stemming from the outlined sources. A secondary component of compassion fatigue per Stamm's conceptualisation is burnout, indicated by perceptions of exhaustion, hostility, and depression. Together, burnout and secondary traumatic stress reflect the negative results of interactions with patients, and this aspect of the working life of nurses has seen great interest in the academic literature based on its ties to several personal and work-relevant outcomes.

The ProQOl is based on the original Compassion Fatigue Self Test (CFS) [8]. In the ProQOL manual, Stamm [9] notes that psychometric problems with the CFS led to a number of revisions resulting in the re-named ProQOL scale. Stamm argues that the ProQOL “addresses difficulties separating burnout and secondary/vicarious trauma” [9]. The ProQOL revision of the CFS reduced the known colinearity between Compassion Fatigue and Burnout. The reported shared variance between Burnout and Compassion Fatigue/Trauma (21%) is described as due to the distress common to both conditions. Despite the acknowledged overlap between the burnout and compassion fatigue/trauma scales, the author states that each of the three scales of the ProQOL (compassion satisfaction, compassion fatigue/secondary traumatic stress and burnout) are psychometrically unique and should not be combined. Further, Stamm acknowledges a complex relationship between the three scales. For example, it is possible for a person to have a high score on both the compassion fatigue and compassion satisfaction scales concurrently.

Stamm’s [1] suggestion that the trigger for compassion fatigue lies with patient interactions has been supported in the nursing literature [10], with traumatic or stressful patient interactions serving to enhance perceptions of compassion fatigue. Hooper, Craig, Janvrin, Wetsel, and Reimels [5] evidenced differences in how compassion satisfaction and fatigue varied between nurses in different caring roles, suggesting elevated scores on compassion fatigue variables for nurses working in intensive care and oncology roles. Burtston and Stichler [11] have provided evidence of variations in nurse caring predicted by nurses’ compassion satisfaction. Ray and colleagues’ [7] recent review of the relationships between compassion fatigue, satisfaction, and work-life conditions for frontline mental health professionals (including nurses) summarised a pattern of previous findings that suggested diminished attitudes or approaches to work in the presence of compassion fatigue. The previously outlined mitigating effect of compassion satisfaction on compassion fatigue was similarly evidenced in their review of the literature [7]. Engagement has previously demonstrated ties to compassion satisfaction and fatigue for nurses [12], such that engagement was negatively associated with nurses’ burnout, although positively associated with compassion satisfaction. Sawatzky and Enns [12] similarly demonstrated that engagement and compassion satisfaction were both significantly correlated with nurses’ intention to leave their positions, a workplace outcome that bears significant financial, social, and knowledge-based costs for employers [13]. Given the ties to patient care, practitioner mental health, employment mobility, and other workplace variables covered in the extant literature (see [7]), the accurate measurement of the constructs encompassed in the ProQOL is arguably an imperative consideration for managers and employers of nurses. However, studies of the ProQOL’s measurement properties have demonstrated limited evidence in this regard, with the key claims to measurement adequacy stemming from the instrument’s author.

Evidence for the validity of the ProQOL, in terms of construct, convergent, and divergent validity, has been arguably underrepresented in the literature. Bride et al. [14] noted that Stamm [9] claimed multi-trait multi-method evidence of convergent and divergent testing of the measure, although reference to the multi-trait multi-method source of evidence is no longer presented in the most recent edition of the manual [1]. While the uncited claim of divergent validity is presented in the current edition of the manual with low correlations between the ProQOL constructs, convergent validity findings remain absent [14]. In terms of construct validity, Stamm [1] described the ProQOL as having “…good construct validity with over 200 published papers”, although the manner in which these papers address construct validity is unclear. Authors such as Watts and Robertson [15] and Bride et al. [14] noted that the construct validity evidence for the ProQOL has not appeared in peer-reviewed literature, and lacked independent verification to the claim of measurement rigour. A key limitation in the construct validity of the ProQOL therefore stems from the limited available information regarding this important factor of measurement. While earlier claims of a lack of factor analysis studies to demonstrate the construct validity of the measure have been presented in the literature (e.g., [7]), recently this appears to have changed with attempts to validate translated versions of the ProQOL. For example, Galiana, Arena, Oliver, Sansó, & Benito’s [16] recent translation of the ProQOL to Spanish and Portugese included tests of construct validity, as did the Hebrew translation validation attempt by Samson, Iecovich, and Shvartzman [17]. In the latter examples however, clear evidence of the measure’s construct validity was not obtained; Samson et al. [17] resorted to exploratory factor analysis after the confirmatory approach failed to find adequate model fit, and Galiana et al. [16] identified problematic construct validity for the burnout latent factor via confirmatory factor analysis. Given the intention of the ProQOL for use as a screening and research instrument, evidence of psychometric rigour is arguably valuable information to have available for practitioners and researchers. To advance this case for measurement rigour further, we propose that an evaluation of this commonly used measure [1] against the principles of measurement, via a Rasch modelling approach, will assist in identifying *how* the ProQOL measures the compassion satisfaction and fatigue of nurses, and whether it supports the intended construct structure outlined by the author.

### Rasch measurement

Rasch measurement [18] represents a philosophically different approach to measurement and instrument validation in comparison to the Classical Test Theory (CTT) approaches that typically dominate the validation literature [19]. To contrast, the CTT approach to validation often aims to formulate a model that best reflects as much variance in collected data as possible. Confirmatory factor analysis and other forms of structural equation modelling are common statistical analysis approaches to this end. Consequently, fitting a model to explain a set of data is the aim of this approach, with the best-case outcome being a model with limited unexplained variance *and* being reflective of a theoretically-meaningful understanding of the latent variable of interest. Rasch measurement places importance on whether the data collected from an instrument’s items provides an invariant representation of an underlying ability or trait of interest. The focus is therefore not on crafting a model or framework that reduces unexplained data variance (and is hopefully theoretically defensible), but for data from measurement items to correctly infer an underlying latent factor [19].

A good understanding of the latent variable of interest to the scale developer and their crafting of the measure items is therefore important, and may undergo several iterations of calibration and refinement, as the items constructed to reflect a latent variable must meet several fundamental measurement requirements per the Rasch model [19]. The underlying factor measured by the items must be singular in nature (i.e., unidimensional), otherwise measurement contamination is inevitable. Bond and Fox [19] provided a good example of early thermometers that varied in their readings on account of temperature and atmospheric pressure, demonstrating a deficiency in accurate instrumentation on the basis of multidimensional measurement. Another key assumption of the Rasch model is that item responses must reflect a linear relationship between the ability or level of a trait a respondent possesses, and their score on the measurement instrument. For example, individuals taking a measure of general mental ability should become progressively less-likely to correctly answer questions of increasing difficulty with respect to how difficult they are in relation to the abilities of the individual. The likelihood (as expressed via a logistic function in the Rasch model) of higher scores on a measure should therefore reflect a linear relationship between individual ability and item difficulty [20]. Items in a Rasch-validated measure must also be invariant in nature. The lack of systematic variation in item responses attributable to facets beyond the underlying factor that the scale is attempting to measure (e.g., the gender of the person providing the response) provides the basis for context-free measurement. These assumptions of the Rasch model represent an exciting approach to instrument validation and refinement in the human sciences, bringing our approaches to measurement closer to that of the fundamental measurement options available to the physical sciences [19].

### Rasch modelling of the ProQOL

Rasch modelling provides several considerations for measure validation, and subsequent refinement, with the stricter measurement principles as outlined prior in comparison to a CTT approach to measure validation. An instrument congruent with Rasch measurement principles has not only theoretical validity-based advantages (e.g., evidence for construct validity), but has practical advantages in affording clear comparisons between scores on a measure (e.g., change scores) due to the interval-level data it can provide [20]. The latter is not possible with traditional summed ordinal-level scoring provided by Likert-style measures, which the ProQOL currently recommends [1], and used in typical CTT approaches to measure validation, although in the latter case this issue is rarely heeded [19]. Additionally, Rasch modelling provides indications of measure reliability that extend beyond the sample-based coefficients typically appearing in the nursing literature, such as the reporting of a Cronbach’s alpha value [20], substantiating validity on the stability of a measure’s item difficulty and person ability ordering. The ProQOL therefore appears to be a valid candidate to this end, due to the ambiguity regarding its measurement properties as outlined prior (e.g., [14]).

There are several questions regarding the response format of the ProQOL that have practical implications for the recommended summed scores approach to interpreting the measure. The Likert-style scale (ranging from ‘Never’ to ‘Very Often’) used by participants completing the ProQOL inherently assumes that participants view each item as being equally difficult to respond to. Realistically, the ProQOL items (e.g., “I am (not) happy” versus “I feel overwhelmed because my case (work) load seems endless”) appear to represent different levels of the underlying variable being addressed, and would therefore be unlikely to encourage participants to consider their responses uniformly. The degree to which participants view items as being more or less likely to be rated highly is an integral part of the Rasch model’s accounting for item difficulty, therefore this aspect of the ProQOL can be scrutinised as part of the planned analysis. It is similarly unclear as to whether all five of the response categories provided to participants are being effectively utilised, or whether certain neighbouring response categories can be collapsed together for reasons of parsimony, which can be established as part of the Rasch analysis procedure. If nurses can convey the same information with fewer response options, then this modification can assist with streamlining the administration of the ProQOL. Furthermore, the recommended 25th and 75th percentile cut-points indicative of low, average, and high levels of CS, STS, and BO outlined by Stamm [1] assume that responses on the ProQOL reflect a well-targeted (i.e., capturing a range of person abilities based on each scales’ items), interval-level measure. This is yet to be demonstrated in the ProQOL literature, and warrants substantiation via Rasch analysis.

Therefore, we seek to examine the measurement properties of the three scales of ProQOL via a Rasch analysis approach. In doing so, we aim to substantiate the construct validity and reliability of the measure, and provide estimates of interval-level normed scores for use in a practitioner setting as a screening tool.

## Method

### Participants

Registered nurses or nurse officers (age x̅ = 46.48 years, *SD* = 11.78) from Australian hospitals participated in the study (*N* = 1615), with the sampled nurses being approximately the same average age as those registered nationally (age *μ* = 44.40 years; [21]). Sampled participants were mostly sourced from public sector (*N* = 664) and private sector (*N* = 651) workplaces, with fewer participants working in the aged care sector (*N* = 273). The sample was predominantly female (*N* = 1360), although the proportion of male nurses (*N* = 116, 7.2%) within the sample was similar to the proportion of male nurses within the occupation broadly (i.e., approximately 10%; [21]). Most of the sampled nurses held less senior roles, with nurses of grade one through three (*N* = 1058) forming the majority of the sample in comparison to more senior nurses of grades four and greater (*N* = 557).

Linacre [22] suggested that, for item calibrations / person measures to be stable within 0.5 logits, at 99% confidence, then a minimum sample size between 108–243 participants (reflective of limited to best targeting, respectively) is required. Our total sample of nurses exceeded these minimum recommended values. Smith et al. [23] have suggested that mean square estimates of fit adequacy are relatively stable across sample sizes for polytomous Rasch models such as the one suggested for this study.

### Measure

#### The Professional Quality of Life scale– 5

The ProQOL consists of three subscales measuring facets of compassion satisfaction and compassion fatigue, targeted at individuals working in caring or helping professions [1]. The three subscales are compassion satisfaction, secondary traumatic stress, and burnout, with the latter two subscales reflecting components of the construct of compassion fatigue [1]. Stamm ([1], [9]) has reported evidence of scale validity and reliability. Based on Stamm’s scale structure, we similarly found good alpha reliabilities for the burnout (*a* = .80), secondary traumatic stress (*a* = .84), and compassion satisfaction (*a* = .90) scales.

### Data analysis

The software Winsteps Version 3.92.1 [24] was used to conduct the Rasch analysis. We examined the adequacy of the items as polytomous partial-credit Rasch models [25] in each instance, to account for the prospect of participants heterogeneously perceiving and responding to the Likert-style scale for each item. Misfitting items were identified on the basis of Infit and Outfit mean-square coefficients outside of the range of 0.60 < *X* < 1.40, as this range reflects reasonable item fit for a Likert-style scale [19]. The reliability of person ordering and item ordering estimates were inferred on the basis of the (more-conservative) real reliability and model reliability and separation coefficients, and Cronbach (KR-20) *α*, calculated as part of Winstep’s model summary output. Larger estimates of reliability were considered reflective of better measurement properties [19]. The univariate structure of the Rasch model was estimated on the basis of a principle components analysis of the Rasch model residuals [19], with off-factor clusters with eigenvalue coefficients greater than 2.0 investigated further for potential issues regarding a multivariate structure [24]. Disattenuated correlations between the residual clusters were similarly examined to help inform decisions made regarding the prospect of a multivariate structure, with disattenuated *r*^*2*^ values greater than 0.50 (i.e., 50% shared variance between the clusters) indicating that a multivariate solution is less likely [24]. Issues with item multicollinearity were detected with standardised residual correlations greater than *r* = .80. Item response category calibrations were made in accordance to the guidelines suggested by Linacre [26] where monotonicity of the response categories, sufficient observations per response category, category Outfit mean-squares coefficients being less than 2.0 were observed. Examination of Differential Item Functioning (DIF) as an indicator of item invariance was conducted on the basis of nurse gender, seniority (nurse rank ≥ 4; nurse rank < 4), and nurse age (50 ≥ years; < 50 years, in addition to X_age_ ≥ years; < X_age_ years). DIF contrast values greater than 0.43, indicative of small-to-moderate effects of differential responding [27], which were statistically significant (*p* < .05) per their corresponding Mantel *χ*^*2*^ coefficient, were considered as evidence of DIF.

### Procedure

The data used in this study is derived from a large-scale survey of nurses conducted in 2013 (see [28] for details). Human research ethics approval was provided by the University of Queensland’s Behavioural and Social Sciences Ethical Review Committee (Approval 2013000887), and by Curtin University’s Human Research Ethics Committee (Approval SONM25-2013). Following human research ethics committee approval, an electronic survey was emailed to members of the Queensland Nursing and Midwifery Union (QNMU) who had a current email address. The survey included a number of structured measures including the ProQOL as well as several demographic questions. An even number of nurses from the public, private and aged care sector were invited to complete the anonymous survey. In total, 13,739 nurses across the three sectors were invited to complete the survey. A total of 2679 (19.50%) nurses completed either some or all of the survey. For the purpose of this study only the nurses who completed the all of ProQOL items were included.

## Results

Descriptive correlation and central tendency coefficients are presented in S1 Table. Starting with the items outlined by Stamm [1] as indicative of each dimension (i.e., burnout, secondary traumatic stress, and compassion satisfaction) of the ProQOL, we conducted a Rasch analysis on each scale to find evidence of a univariate latent structure for each construct, monotonically ascending response categories, and invariant item responding based on nurse demographic variables.

### Compassion satisfaction

#### Person and item fit

The initial Rasch model estimation of the Compassion Satisfaction (CS) scale suggested that nurses found the scale items easy to endorse, as evidenced by the positive person-measure estimate (1.76, Model *SE* = .55). Estimates of person reliability appeared to indicate good fit (real reliability estimate (real) = .85; model reliability estimate (model) = —.89), which was supported by excellent person separation estimates (real = 2.39; model = 2.79). Furthermore, the CS scale demonstrated excellent Cronbach reliability (*a* = .92). Estimates of item reliability were excellent (0.99; separation real = 10.45; model = 10.78). Examination of Infit and Outfit mean-square estimates for items did not demonstrate evidence of notable misfit suitable for a Likert-style scale (.60 ≤ X ≤ 1.40; [19]) for any items, and all items demonstrated good partial correlations with the overall measure (*r* > .67) in the same direction. We confirmed a univariate latent factor structure underlying the model via a principle components analysis of the model residuals, which indicated that the largest cluster of residuals had an Eigenvalue < 2.0, and was therefore unlikely to indicate multidimensionality [24]. Dependency in participant responses for items was not evident, as all items had standardised residual correlations *r* < .70, suggesting non-redundancy among items [20]. Following these initial checks of model adequacy, we then examined the adequacy of each item’s response categories as a means of improving the model’s targeting, followed by checks for measurement invariance across participant gender, age, and nurse grade.

#### Item response categories

When examining the monotonicity of the item response categories, responses from participants in the ‘Never’ and ‘Rarely’ categories of the five-point Likert-style scale suggested a potential need for revision for many of the CS items. We conducted revisions of the items per the guidelines for category collapsing outlined by Linacre [26], collapsing the first two (‘Never’ and ‘Rarely’) and three (‘Never’, ‘Rarely’, and ‘Sometimes’) response categories for items where improvements in observations per category, category fit, and coherence were noted. Items 6, 16, 18, 20, 22, and 30 were recoded to allow the first two response categories to be collapsed. Items 3, 12, 24, and 27 were recoded to allow the first three response categories to be collapsed. This revised recoding of the CS items prompted an improvement in person measure targeting (0.70, *SE* = 0.60; Initial = 1.76, *SE* = 0.55) to better reflect the level of average nurse compassion satisfaction. The person-level reliability (real = .87; model = .89), separation (real = 2.58; model = 2.90), and Cronbach reliability (*a* = .90) remained excellent following the response category recoding. The item-level reliability estimates (1.00), and separation (real = 14.19; model = 14.59) remained excellent. We therefore continued with our further examination of the CS scale as a measure befitting of three or four response options, depending on the CS item being measured.

#### Invariance testing

We examined measurement invariance across factors of age, gender, and nurse grade seniority. Consistent with the Australian Institute of Health and Welfare’s [21] contrasting of nurses on either side of the 50 years of age threshold, we examined measurement invariance on the CS scale items for nurses older or younger than 50 years of age. Additionally, we examined age-related invariance via splitting nurses on the basis of being younger or older than the mean age of the sample (*X*_*age*_ = 46.48 years). Neither method of examining invariance across age groups demonstrated variant response characteristics, as all contrasts demonstrated statistically non-significant Mantel *χ*^*2*^ coefficient probabilities and/or differential item functioning (DIF) contrasts < 0.43 logits, the latter indicating a non-meaningful proportion of item variance across demographic categories ([24], [27]). No evidence of DIF was similarly presented based on the gender of nurses. We conducted our final test of invariant responding on the CS scale based on the grade seniority of the nurses, contrasting registered nurses (or nurse officers) at grades 1 through 3, to those at grade 4 or greater. We found no evidence of invariant responding when contrasting nurse grades along those categories. In summary, the CS scale of the ProQOL, with its modified response scoring approach, appeared to demonstrate good item fit (see Table 1), and invariant responding across several demographic characteristics. We present in Table 2 interval-level scores for the CS scale based on the modified scoring format, and Table 3 presents the 25^th^, 50^th^, and 75^th^ percentile cut-point equivalents to Stamm [1] using our modified scoring format. Table 4 presents bivariate correlations and coefficients of central tendency between the modified scoring measure items and the summed measure scores representing CS.

**Table 1:** Item measure and fit coefficients, point-correlation with measure coefficients, and coding reference for reduced ProQOL-21 item set.

ProQOL Item	Measure	Model *SE*	Infit Mnsq	Outfit Mnsq	Pt. *r*	Coding
Compassion Fatigue						
26 (Male)	-1.89	0.19	1.01	0.93	0.73	12233
26 (Female)	-1.33	0.06	1.08	1.19	0.62	12233
21	-1.26	0.04	1.34	1.34	0.70	12345
19	-1.24	0.04	1.21	1.22	0.72	12345
23	0.01	0.05	1.13	1.24	0.58	12333
10	0.17	0.04	0.92	0.90	0.69	12344
11	0.24	0.04	0.73	0.75	0.74	12344
9	0.40	0.04	0.91	0.93	0.74	12345
8	0.51	0.05	1.00	1.14	0.64	12344
14	0.85	0.05	0.90	0.85	0.66	12344
25 (Male)	0.94	0.18	0.86	0.69	0.71	12344
13	1.24	0.05	0.88	0.90	0.70	12345
25 (Female)	1.37	0.06	0.86	0.67	0.60	12344
Compassion Satisfaction						
30	-0.96	0.05	0.92	0.90	0.75	11234
3	-0.73	0.06	0.9	0.91	0.71	11123
22	-0.44	0.05	0.95	0.93	0.77	11234
16	-0.19	0.05	1.14	1.15	0.72	11234
20	-0.19	0.05	0.93	0.92	0.77	11234
24	-0.01	0.06	0.76	0.72	0.77	11123
18	0.06	0.05	1.05	1.07	0.75	11234
6	0.17	0.05	1.33	1.35	0.68	11234
12	0.21	0.06	0.93	0.93	0.73	11123
27	2.06	0.06	1.09	1.12	0.69	11123

Measure = item difficulty relative to the underlying factor. Mnsq = Mean-square estimate. Pt. *r* = Point-correlation with measure. Coding = Modification of the original scoring approach (i.e., 12345 for original responses ‘Never’, ‘Almost Never’, ‘Sometimes’, ‘Often’, and ‘Very Often’, respectively) to reflect the collapsed response categories where appropriate.

**Table 2:** Raw scores and corresponding normed scores, standard errors, and percentile rankings for the modified ProQOL-21 scales.

	Compassion Satisfaction		Compassion Fatigue (Female)		Compassion Fatigue (Male)	
Raw Score ^a^	Normed Score (SE)	%	Normed Score (SE)	%	Normed Score (SE)	%
10	177 (87)	1				
11	237 (50)	1	235 (104)	2	290 (89)	4
12	276 (37)	1	308 (60)	5	352 (51)	9
13	301 (32)	2	356 (45)	8	392 (38)	13
14	322 (30)	3	388 (39)	12	418 (32)	17
15	340 (28)	5	413 (36)	18	437 (29)	23
16	357 (27)	7	435 (34)	24	454 (27)	26
17	373 (27)	10	454 (32)	29	468 (25)	30
18	388 (26)	13	471 (30)	34	481 (24)	36
19	402 (26)	17	487 (29)	40	492 (23)	43
20	416 (25)	20	501 (28)	46	502 (22)	49
21	430 (25)	24	515 (27)	52	512 (21)	53
22	444 (25)	27	527 (26)	58	521 (20)	58
23	457 (25)	32	539 (25)	64	529 (20)	66
24	470 (25)	38	549 (24)	68	537 (19)	71
25	484 (25)	44	560 (24)	73	545 (19)	74
26	497 (25)	50	570 (23)	76	552 (18)	76
27	511 (26)	56	580 (23)	80	559 (18)	77
28	526 (26)	61	589 (23)	83	566 (18)	78
29	541 (27)	67	598 (23)	86	573 (18)	80
30	556 (27)	74	607 (23)	88	580 (18)	81
31	573 (30)	79	616 (23)	90	587 (18)	82
32	591 (30)	83	625 (23)	92	594 (18)	84
33	612 (33)	88	635 (23)	93	601 (19)	86
34	638 (38)	92	644 (23)	95	609 (19)	87
35	677 (50)	95	653 (23)	96	617 (19)	89
36	738 (87)	98	663 (24)	97	625 (20)	91
37			674 (24)	97	634 (21)	92
38			685 (25)	98	643 (21)	93
39			697 (26)	98	653 (23)	95
40			709 (28)	99	665 (24)	97
41					678 (26)	97
42					693 (28)	98
43					711 (32)	98
44					736 (38)	99

Normed scores have a mean of 500, with a standard deviation of 100. Blank cells indicate values that are out of range based on each scale’s potential minimum and maximum raw score. % = Percentile.

^a^ Raw scores based on the modified scoring recommendations provided in-text, see the Coding column in Table 1 for recoding specification. Note that for the Compassion Fatigue scales, higher raw scores than indicated in this Table are possible (i.e., Maximum = 46), but are similarly reflective of individuals at the 99^th^ percentile, and are therefore not reproduced due to redundancy.

**Table 3:** Percentile cut-points for modified ProQOL-21 scoring approach.

Percentile	Compassion Satisfaction	Compassion Fatigue (Female)	Compassion Fatigue (Male)
25th	21	16	15
50th (*μ*)	26	20	20
75th	30	25	25

The scores used to infer cut-points are based on scale totals following the modified scoring approach detailed in the Coding column in Table 1.

**Table 4:** Bivariate correlations and coefficients of central tendency for modified ProQOL-21 scoring approach items.

Item	3CS	6CS	12CS	16CS	18CS	20CS	22CS	24CS	27CS	30CS	8BO	9STS	10BO	11STS	13STS	14STS	19BO	21BO	23STS	25STS	26BO	CS Total	CF Total
6CS	.527**																						
12CS	.576**	.457**																					
16CS	.440**	.424**	.494**																				
18CS	.464**	.407**	.516**	.488**																			
20CS	.536**	.486**	.517**	.506**	.537**																		
22CS	.522**	.428**	.527**	.501**	.549**	.567**																	
24CS	.575**	.469**	.553**	.522**	.538**	.596**	.652**																
27CS	.399**	.411**	.427**	.476**	.449**	.468**	.516**	.537**															
30CS	.533**	.432**	.600**	.507**	.621**	.547**	.541**	.584**	.428**														
8BO	-.138**	-.084**	-.171**	-.187**	-.178**	-.133**	-.164**	-.146**	-.113**	-.173**													
9STS	-.100**	-.067*	-.158**	-.209**	-.179**	-.149**	-.122**	-.105**	-.065*	-.169**	.583**												
10BO	-.341**	-.267**	-.402**	-.326**	-.396**	-.305**	-.318**	-.337**	-.242**	-.447**	.472**	.541**											
11STS	-.215**	-.162**	-.266**	-.257**	-.285**	-.220**	-.234**	-.243**	-.155**	-.288**	.540**	.634**	.704**										
13STS	-.110**	-.097**	-.136**	-.205**	-.204**	-.163**	-.176**	-.157**	-.145**	-.187**	.528**	.600**	.492**	.587**									
14STS	-.108**	-.092**	-.163**	-.178**	-.170**	-.124**	-.128**	-.140**	-.106**	-.168**	.549**	.548**	.477**	.521**	.708**								
19BO	-.157**	-.173**	-.216**	-.254**	-.283**	-.251**	-.199**	-.166**	-.137**	-.298**	.332**	.427**	.471**	.472**	.398**	.362**							
21BO	-.149**	-.180**	-.216**	-.246**	-.288**	-.212**	-.195**	-.158**	-.132**	-.236**	.350**	.391**	.399**	.429**	.379**	.341**	.599**						
23STS	-.170**	-.116**	-.232**	-.185**	-.201**	-.174**	-.175**	-.214**	-.144**	-.168**	.443**	.422**	.388**	.432**	.452**	.488**	.298**	.301**					
25STS	-.204**	-.170**	-.209**	-.251**	-.231**	-.194**	-.176**	-.210**	-.150**	-.223**	.479**	.480**	.505**	.559**	.524**	.533**	.364**	.349**	.542**				
26BO	-.176**	-.147**	-.211**	-.245**	-.379**	-.247**	-.231**	-.207**	-.125**	-.312**	.291**	.378**	.450**	.437**	.317**	.279**	.500**	.526**	.257**	.376**			
CS Total	.731**	.681**	.749**	.718**	.757**	.769**	.779**	.803**	.675**	.783**	-.194**	-.174**	-.453**	-.313**	-.217**	-.188**	-.280**	-.265**	-.234**	-.261**	-.298**		
CF Total	-.239**	-.203**	-.305**	-.330**	-.356**	-.278**	-.269**	-.265**	-.195**	-.342**	.702**	.770**	.757**	.809**	.758**	.727**	.696**	.676**	.621**	.707**	.614**	-.370**	
*M*	2.370	2.728	2.166	2.837	2.757	2.801	2.925	2.226	1.690	3.146	1.674	2.147	1.796	1.808	1.787	1.596	2.912	2.880	1.576	1.382	1.999	25.624	21.546
*SD*	0.661	0.897	0.748	0.845	0.875	0.815	0.874	0.741	0.726	0.895	0.834	0.952	0.956	0.907	0.874	0.790	1.104	1.131	0.721	0.712	0.692	6.006	6.917

Bivariate correlations reflect Pearson’s *r* coefficients. Item numbers reflect numbering from ProQOL instrument manual [1]. CS = Compassion Satisfaction. BO = Burnout. STS = Secondary Traumatic Stress. *M* = sample mean, *SD* = standard deviation.

** *p* < .01.

### Burnout and secondary traumatic stress

Our initial examination of the burnout and secondary traumatic stress measures indicate notable limitations in measurement adequacy (see S1 Results). We found that the burnout measure indicated the best-fitting model when considered as a two factor measure; a consequence of the limited item pool in each minor scale was mediocre reliability and measure targeting. Similarly, a reduction in items for the secondary traumatic stress scale after consulting evidence of item-misfit, alongside evidence of mediocre targeting and 60% of the scale indicating concerns with differential item functioning, suggested that the item-factor relationships proposed by Stamm [1] were difficult to support with our analyses. Returning to the pool of twenty items from the burnout and secondary traumatic stress scales, we tested the prospect of the ProQOL demonstrating adequate measurement properties for a latent compassion fatigue factor, reflected by items from both scales. This approach is consistent with Stamm’s [1] description of Burnout (BO) and Secondary Traumatic Stress (STS) reflecting a general construct of Compassion Fatigue (CF), or the negative consequences of caring for clients, therefore we considered this revised approach theoretically justifiable.

#### Dimensionality

**Fig 1:**
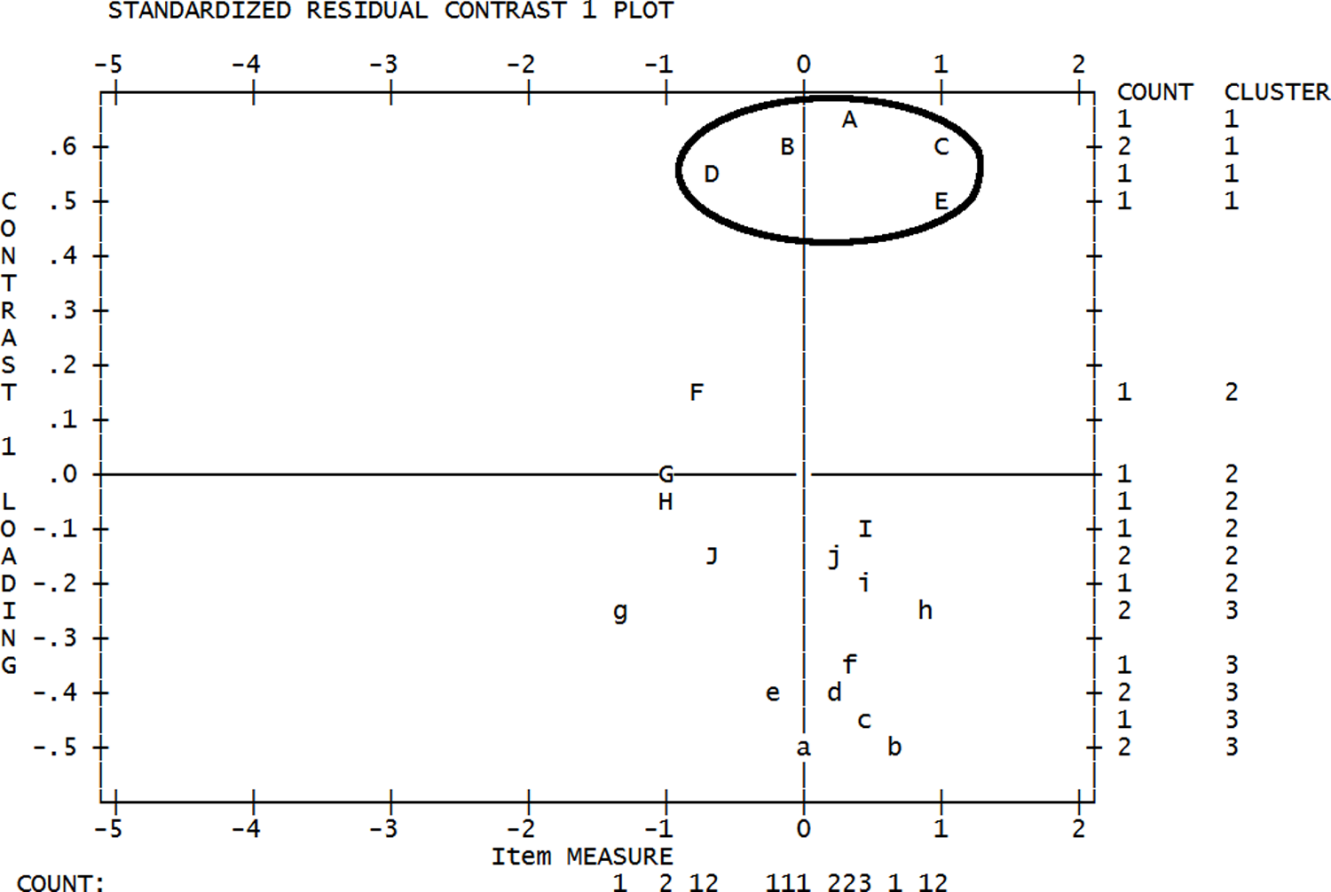
Principal components analysis standardised residual plot for compassion fatigue measure. Standardised residual contrast plot indicating a five item cluster (circled) with notable separation along the Y axis from the remaining Compassion Fatigue items.

After inclusion of the 10 Burnout and 10 Secondary Traumatic Stress items, we conducted an initial inspection of dimensionality to support the proposed singular CF underlying construct, before considering item fit, invariance, and other considerations of measurement adequacy. While the raw variance explained by the measures was less than 50% (Eigenvalue = 16.24, 44.8% variance), this appeared to be muddied by a substantial off-factor cluster of items as suggested by a notable first contrast (Eigenvalue = 3.04, 8.4% variance). This cluster of items consisted of BO scale items, specifically items 4, 17, 29, 15, and 1 (per the item ordering in [1]). We consulted the disattenuated correlations between this cluster (see Fig 1) and the remaining two clusters, and noted that these items appeared to have very little shared variance with the remaining items from the BO and STS scales; *r =* .429 (*r*^*2*^ = .184, or 18.4%) with the second cluster, and *r* = .288 (*r*^*2*^ = .083, or 8.3%) with the third cluster. Due to the limited shared variance of these items, insinuating they were unlikely to represent the same underlying factor as the remaining 15 items, we removed these items and re-examined dimensionality.

A second test of dimensionality among the remaining 15 items from the secondary traumatic stress and burnout scales indicated a potentially-significant off-factor cluster (Eigenvalue = 2.19, 6.7% of variance). This cluster consisted of items 21, 26, and 19 from Stamm’s [1] burnout subscale, although in this instance the disattenuated correlations between this cluster and the remaining clusters did not seem to indicate multidimensionality as prominently. The relationship between clusters 1 and 2 (*r* = .682, *r*^*2*^ = .465, or 46.5% shared variance) and clusters 1 and 3 (*r* = .793, *r*^*2*^ = .629, or 62.9% shared variance) suggested that near-half or greater than half of the variance between the clusters was shared, respectively. Consequently these items did not seem to be clear candidates for removal from the model at this stage of analysis, and were retained as indicators of CF. The overall quantity of raw variance in the underlying construct explained by the measure at this stage of analysis was over 50% (Eigenvalue = 17.49, 53.8% variance explained).

#### Item fit and item response categories

The initial Rasch model, consisting of the 15 items retained that reflected CF, indicated that nurses found the items generally difficult to endorse on the basis of its negative person-measure coefficient (-1.20, *SE* = 0.37). Person reliability (real = .84; model = .88) and separation (real = 2.30; model = 2.67) coefficients suggested that the items demonstrated adequate person-reliability, which was corroborated by Cronbach’s (KR-20) alpha reliability, *a* = .90. Item reliability (real/model = 1.00) and separation (real = 20.24; model = 21.18) coefficients indicated no concerns with item-reliability. We conducted item response category calibration next, and the collapsing of redundant response categories indicated that the CF items were best-reflected by 3, 4, or 5 response categories (see Table 1).

While item response category calibration drew the measure’s targeting closer to the midpoint of zero (-0.97, *SE* = 0.40) and consolidated the monotonically-ascending nature of the items’ rating scales, issues with item-misfit for three items were noted on the basis of high (>1.40; [19]) Infit and Outfit mean-square coefficients. Item 2 of the STS scale “I am preoccupied with more than one person I (help).” [1] demonstrated substantial Infit/Outfit coefficients (1.49/1.63, respectively). These values suggested the item was capturing substantial variance outside of the underlying CF factor, and performing poorly as a predictor of CF at the estimated level of item difficulty. Examination of the overlaid empirical and expected item-characteristic curve output suggested that nurses at lower levels of CF were responding with higher scores on this item than predicted based on the calculated 95% confidence intervals (i.e., scores were outside the 95% CI bands), while nurses at higher levels of compassion fatigue were scoring lower than anticipated based on a similar examination of the 95% CI bands. As we viewed the item to reflect a common element of nursing work (i.e., considering the needs of multiple patients) instead of CF specifically, the poor performance of this item appeared to be logically reasonable, and we therefore removed it from subsequent analysis. Items 28 (“I can't recall important parts of my work with trauma victims”; Infit = 1.33, Outfit = 1.58) and 5 (“I jump or am startled by unexpected sounds”; Infit = 1.54; Outfit = 1.57) [1] appeared to capture substantial variance outside of CF. Consultation of the empirical and expected item-characteristic curve output suggested that both items encountered difficulty with nurses predicted to have higher levels of CF, as the responses on these items for nurses with higher CF was notably less than anticipated, and outside of the 95% CI bands predicted by the model. Scrutiny of the item content of these items appeared to support the analysis’ findings of accounting for variance beyond CF. For example, lapses in recall may reflect outside factors, such as the varying levels of trauma-patient knowledge that nurses may have due to experiential differences [29], while being startled by unexpected sounds did not reasonably seem to be a behaviour demonstrated by nurses with compassion fatigue alone (i.e., the generic acoustic startle reflex; [30]).

Following the removal of these two items, an additional potentially misfitting item was noted on the basis of its mean-square Outfit coefficient (1.44). Item 7 (“I find it difficult to separate my personal life from my life as a (helper)”), based on item-content, appeared to be potentially tapping into outside constructs such as the perception of work-life balance held by the nurse, a construct that is influenced by an array of environmental and individual facets [31]. Additionally, the empirical and expected item-characteristic curve output suggested that this item did not capture the scores of nurses high in CF adequately, finding instead that nurses high in CF appeared to rate this item notably lower than anticipated, placing these scores outside of the 95% CI boundaries predicted by the model. Consequently the high Outfit mean-square coefficient of this item appeared to be a reasonable indication of an item with off-factor measurement, and it was subsequently withheld from further analysis of the measure.

The remaining 11 items demonstrated sufficiently well-fitting mean-square Infit and Outfit coefficients (i.e., Infit/Outfit 1.40 > X > 0.60; [19]). Additionally, they retained acceptable person-reliability (real = .84; model = .88) and person-separation (real = 2.33; model = 2.69), item-reliability (1.00) and separation (real = 21.10; model = 21.72), and Cronbach’s (KR-20) alpha reliability, *a* = .90. The measure’s person-targeting remained -1.37 logits (*SE* = 0.53) off from the ideal zero-value however, suggesting that the CF items retained were difficult for the average participant to endorse (i.e., indicate higher ratings on the Likert-style scales of the items). Table 4 presents bivariate correlations and coefficients of central tendency for the items retained for the CF measure. The revised-scoring procedure CS and CF measure scores correlated significantly and negatively, *r* = -.370, *p* < .01.

#### Invariance

**Fig 2:**
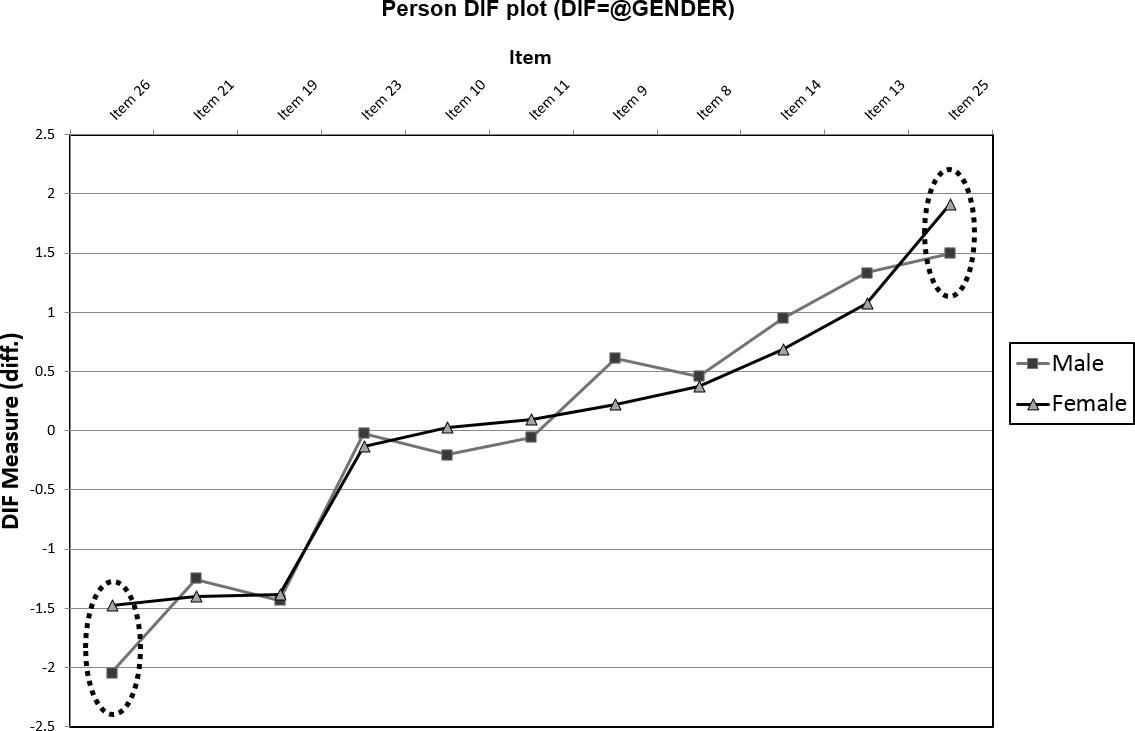
Person differential item functioning (DIF) plot for compassion fatigue items. Significant (Mantel *χ*^*2*^ *p* < .05) and notable (DIF contrast coefficient > 0.43; [27]) gender-variant responding was identified. Items 25 and 26 (dotted-circled) had significant, notable differences in item-difficulties between male and female nurse respondents.

As item fit, calibration, and unidimensionality were reasonably justified for the CF measure, tests of item invariance across gender, seniority, and age categories were conducted. While nurse seniority and age categories did not indicate potential concerns with variant responding, examining the differential item functioning (DIF) of the CF items on the basis of nurse gender suggested invariant responding (see Fig 2) for item 26, “I feel ‘bogged down’ by the system” [1]. Male nurses (item difficulty = -2.05 logits) found item 26 significantly easier to endorse in comparison to their female counterparts (item difficulty = -1.47; Mantel *χ*^*2*^ = 4.54, *p* = .033, DIF contrast = 0.57 logits). As this degree of difference represented a slight-to-moderate level of invariant responding ([24], [27]), we created a male-response and female-response version of item 26, before adding these items to the model after removing the general-response version of item 26, as a means of accounting for the DIF of this item ([24], [32]). Following the introduction of these items, item 25 (“As a result of my (helping), I have intrusive, frightening thoughts”; [1]) demonstrated notable DIF between male (item difficulty = 1.62) and female (item difficulty = 2.08; Mantel *χ*^*2*^ = 5.70, *p* = .017, DIF contrast = 0.46 logits) nurses. Male nurses did not perceive this item as difficult to endorse as female nurses, and this item was reflective of a small-moderate degree of differential item functioning ([24], [27]). Splitting item 25 into gender-variant versions using the same approach outlined prior did not reveal any further evidence of DIF. Table 1 presents the item difficulties, recoding modifications, and fit coefficients, and we present in Table 2 the interval level scoring of the final modified CF scale. Table 3 provides cut-points per Stamm’s [1] 25^th^, 50^th^, and 75^th^ percentile delineations.

## Discussion

In examination of the measurement properties of the ProQOL [1] via a Rasch modelling approach, the measure demonstrated mixed findings. Overall, we found support for the construct validity of the compassion satisfaction scale of the ProQOL. However, the secondary traumatic stress scale and burnout scale did not demonstrate evidence of satisfactory construct validity with the current set of items in Stamm’s [1] instrument. We provided an alternative argument for Stamm’s [1] burnout and secondary traumatic stress items as being reflective of a theoretically-justifiable measure of compassion fatigue, reinforced by good item and model fit evidence. Per the recommendations of Bond and Fox [19], the initially mediocre burnout and secondary traumatic stress measures were not an impetus for us to ‘throw out’ the items altogether; instead, we repurposed these items as reflecting the trauma and burnout indicators of compassion fatigue.

The CS scale demonstrated invariant responding on its items, and reflected a univariate latent factor driving the nurses’ responses on its items. The overall person-level targeting of the CS scale suggested that nurses found the items generally too easy to endorse however (person targeting measure = 0.70, *SE* = 0.60), with prominent “gappiness” in the distribution of items for nurses higher in compassion satisfaction (see Table 1). Furthermore, the response categories for the CS scale suggested that the Likert-style scale’s five response options were difficult for nurses to separate meaningfully. Our analysis indicated that four or three response categories were meaningful for nurses on the CS items, with the Strongly Disagree and Disagree responses being effectively synonymous in meaning for the nurses on the CS items. Our condensed response category structure outlined in Table 1 therefore reflects a more-parsimonious and robust approach to scoring participant responses in comparison to Stamm’s [1] recommended default approach. The person reliability and item reliability indices for the recalibrated CS scale were good (>.80, Cronbach *a* = .90), which reflected the well-fitting properties of the items.

The construct validity of the CS scale was largely supported by our findings, which was unfortunately in contrast to our attempt to validate the burnout and secondary traumatic stress scales of the ProQOL. Our resultant CF scale, drawing from the burnout and secondary traumatic stress items to retain items that loaded meaningfully on a univariate latent factor of compassion fatigue, was a solution to the mediocre scale properties identified during our attempt to validate the scales separately. The resultant measure contained items that were difficult for nurses to endorse (person targeting measure = -1.37, *SE* = 0.53), suggesting that items that reflected lower-levels of compassion fatigue were not sufficiently represented in the final 11 items of our CF scale. In a similar vein to that of the CS scale, item response categories were recalibrated for the CF items to better-fit the degree to which participants could meaningfully discriminate between scale response options. Negatively-worded items unique to the burnout scale in Stamm’s [1] ProQOL all indicated poor fit with the underlying compassion fatigue factor, and were subsequently removed. This outcome appeared to be consistent with the cautions presented by Bond and Fox [19] regarding negatively-worded items. Bond and Fox [19] suggested that negatively-worded items are likely to be understood differently by participants in comparison to positively-worded items, and may therefore misfit with the latter regardless of whether algebraic reversing of scores has been performed on these items. Furthermore, two items in the CF scale demonstrated response variance dependent on the nurse’s gender, such that male nurses perceived these items as easier to endorse in comparison to their female peers (see Tables 1 and 3). In light of these measure weaknesses, a univariate solution was confirmed for the CF scale, as was evidence of adequate item-fit for the retained items, and adequate person reliability and item reliability indices (>.80, Cronbach *a* = .90). Both the CS and CF scales are therefore argued to demonstrated rigorous evidence of construct validity, following the modifications outlined prior. Consequently, this finding raises considerable questions about the construct validity of the burnout and secondary traumatic stress scales presented by Stamm [1].

### Theoretical implications

Our findings suggest several important theoretical implications for the use of Stamm’s [1] ProQOL, particularly within the profession of nursing. Primarily, the difficulty in substantiating the validity of the ProQOL’s burnout and secondary traumatic stress items as distinct, well-functioning scales is a point of concern given the measure’s use in healthcare practitioner research (e.g., [4], [5], [6]). Particularly in instances where research has been conducted using the burnout or secondary traumatic stress scales as indicators of their inferred latent factors (e.g., [2]), the adequacy of citing an alpha coefficient and the validity claims presented in the measure’s manual are potentially questionable forms of evidence. Regarding the former, alpha is frequently and incorrectly regarded as an indicator of unidimensionality/homogeneity, and widely-cited “cut-off” values such as *a* > .70 are not guarantees of measure reliability [33], therefore alpha coefficients demonstrated by previous research using the ProQOL do not dismiss this validity query. Instead, this finding reinforces questions raised by authors such as Bride et al. [14] and Ray et al. [7] about the limited representation in the literature regarding the measure’s construct validity, substantiated by our mixed evidence to this end. Researchers working with data collected from the ProQOL may wish to use our revised ProQOL-21 item and scoring approach (see S1 Syntax for SPSS Syntax) if they wish to make inferences about compassion satisfaction and compassion fatigue. If researchers intend to examine relationships with burnout and secondary traumatic stress as inferred by the ProQOL, we caution that this approach may require further clarification from future literature to confirm its tenability, specifically regarding evidence of construct validity for these measures.

Echoing the advice provided by authors such as Linacre [24] and Bond and Fox [19], our findings do not necessarily indicate that the items provided by Stamm [1] are unsalvageable. Despite the limitations in accounting for noteworthy off-factor variance, and/or poorly reflecting participant ability at the estimated level of item difficulty, Bond and Fox [19] recommend that findings such as these serve as a signal to researchers to “…‘find out why’ not ‘throw it out’”. As we noted in the Results, many of the predictors we set aside in this iteration of analysis appeared to account for variance outside of compassion fatigue. While we have attempted to provide contextual explanations as to why scoring on these items did not conform to what was expected (i.e., an assumed underlying compassion fatigue factor driving participant responses), the concepts behind the original item generation by Stamm [1] may still be valid, albeit requiring some rewording or further clarity enhancement to better reflect facets of the intended underlying factor. Take, for example, item 5 (“I jump or am startled by unexpected sounds”; [1]). While this item presumably sought to reflect the increased arousal experienced by healthcare professionals with secondary traumatic stress [34], we argued that it was likely to also capture variance associated with the acoustic startle reflex [30]. Therefore an alternative item, intended to address the facet of increased physiological arousal argued to be reflective of individuals experiencing secondary traumatic stress, is prospectively a valid future focus for an addition to the original ProQOL items. It is with this in mind that we note that the conceptualisation of burnout and secondary traumatic stress are not inherently flawed, a stance that is reinforced by the sound reasoning of previous authors in this field. Instead, the ProQOL requires further iterations to address the paucity of well-fitting items [19] relevant to the burnout and secondary traumatic stress subscales. Therefore, for the purposes of theory generation and model testing, we would recommend that authors intending to measure the latent factors of burnout and secondary traumatic stress consider alternative available measures (e.g., the short burnout measure of [35]) until a future item-generation iteration of the ProQOL has been calibrated and validated.

In terms of theory-generation relevant to substantiating a general Compassion Fatigue construct, our investigation of the construct validity of the ProQOL indicates that a mix of items originally from the burnout and secondary traumatic stress (STS) scales, when combined, produces a valid, short-length measure of compassion fatigue. Rather than separating burnout and STS as separate constructs, the present analysis indicates that the construct is better captured as a combination of STS items that capture aspects of the trauma associated with caring, along with other items related to emotional and physical exhaustion associated with caring. Stamm [9] suggested that the overlap between the burnout and secondary traumatic stress scales was due to distress common to both conditions. Our results suggest that rather than two independent but related conditions as proposed by Stamm [9], compassion fatigue can be meaningfully construed as one construct, which may assist researchers examining broader negative facets of healthcare provision, and their anticipated outcomes.

### Practical implications

The reduced number of items for the revised ProQOL-21 lends itself to greater ease of administration in a practitioner setting as a screening tool, per Stamm’s [1] recommended use of the scale. Both the compassion satisfaction and compassion fatigue measures presented in our Results demonstrated excellent measurement characteristics, therefore practitioners can confidently apply our revised approach to scoring and inference as part of their intended use as a screening tool. In both clinical and research settings this brief version of the ProQOL means that it is possible to measure nurse professional quality of life in a more efficient and less time-consuming manner. This is important, as nurses are often asked to complete many workplace surveys, therefore a shortened version of this measure can reduce the prospect of being over-burdened in this regard.

We are aware that the ProQOL has been and continues to be used extensively around the world [1]. In order to use this existing pool of data, we have developed an SPSS syntax file available to other researchers and practitioners (see S1 Syntax) to re-score their ProQOL data according to the two scale structure reported here. This will enable researchers and practitioners to determine a total score for CS and CF that can then be compared with data collected by different research groups across different settings.

### Limitations

Our male sample (*N* = 116) was considerably fewer than our female sample, which may have had implications for the estimation of error terms for this subsample. Future studies examining the measurement properties of the ProQOL should try to sample additional male participants, as it would assist in bolstering the confidence of accuracy for the measure’s estimations within this minority group of nurses. While the low response rate in this study is common in nursing research [36], the potential issue of participant bias in participating nurses may be a prospective limitation of this research. To temper this prospect, we note that the demographic characteristics of the nurses participating in this study were highly similar to those reported by national data on employed nurses [21], therefore biased participant selection was not a clear threat to these findings.

### Directions for future research

The results of this study open up many avenues for future research. As noted previously, for the ProQOL to be regarded as a valid measure of burnout and secondary traumatic stress in particular, further research must be directed to iterating additional items that address the notable gaps and measurement shortfalls for these subscales. However, should future researchers wish to examine compassion satisfaction and compassion fatigue, our revised scoring procedures (which is further facilitated with the available SPSS syntax in S1 Syntax) provide a rigorous footing with which to explore these constructs as indicated by the existing ProQOL item set. Studies of this nature are essential steps in the creation of theory-driven interventions aimed at building resilience and reducing compassion fatigue [2]. To this end, two important directions for future research appear necessary regarding the modified version of the ProQOL. Due to the use of Australian participants in this study, the potential for differential item functioning on the basis of cultural differences could not be explored. As previous studies (e.g., [16]) have noted intercultural variations when examining the factor properties of the ProQOL, it would be valuable for research conducted in other countries to confirm the adequacy of the measure properties as part of the iterative measure refinement process we propose. Furthermore, while the construct validity of the measure was the focus of inquiry in this article, other facets of validity (e.g., predictive validity) merit exploration. While we have presented fundamental bivariate correlation coefficient information to demonstrate the revised-scoring procedure measures’ negative relationship with each other as anticipated in the CS/CF literature [1], future research may wish to examine predictive validity via other variables (e.g., resilience; [2]) following the measure modifications.

The use of the partial-credit Rasch model in our analyses, serving to maximise the model’s person-targeting characteristics, has implications for the presentation of the ProQOL test items and their response options. While a variable series of response anchors and response choice lengths may have advantages in reducing acquiescent-responding biases [37], we are concerned about the potential for this to be a clarity-inhibiting element in scale presentation. Therefore we recommend practitioners and researchers employ post-data-collection recoding to adjust for the category response limitations highlighted in our analyses. Additional items may allow future iterations of this measure to apply a consistent scoring approach across all items without the targeting limitations outlined in our Results (i.e., demonstrate good targeting via a rating scale model). In addition to addressing item-difficulty gaps and item misfit issues by generating additional items for the ProQOL, and in-turn validating these items are indicators of a valid measurement model, a future iteration of the measure may be able to uniformly present a well-targeted series of response options without needing to have item-level variations in response category behaviour.

## Conclusions

Our findings present measures of compassion satisfaction and compassion fatigue based on Stamm’s [1] ProQOL items, which we have dubbed the ProQOL-21. Both measures demonstrated excellent person- and item-fit characteristics, and fulfilled the strict requirements of Rasch measurement models. We therefore recommend researchers and practitioners consider using these measures based on the presented evidence of excellent measurement adequacy and scale brevity.

## Supporting information

S1 Table Bivariate correlations and coefficients of central tendency for ProQOL items. Bivariate correlations reflect Pearson’s r coefficients. Item numbers reflect numbering from ProQOL instrument manual [1]. CS = Compassion Satisfaction. BO = Burnout. STS = Secondary Traumatic Stress. *M* = sample mean, *SD* = standard deviation. * *p* < .05. ** *p* < .01.
(DOCX)

S1 Results Analyses of the burnout and secondary traumatic stress scales considered individually. This set of supplementary results details the attempts at examining whether the Burnout and Secondary Traumatic Stress measures in the ProQOL conformed to the assumptions of a Rasch model.
(DOCX)

S1 Syntax SPSS statistics recoding syntax for the modified ProQOL-21 scales. To assist users of SPSS Statistics with transforming the coding of their existing ProQOL datasets to employ the revised response categories and subscale scores outlined in this article, this syntax file recodes items and provides subscale scores accordingly. Note that the syntax is provided as-is for researchers and practitioners to use, and we recommend that users should retain a copy of their original data prior to recoding.
(SPS)
