# Supplementary material for: The ProQOL-21: A revised version of the Professional Quality of Life (ProQOL) scale based on Rasch analysis
Source: PLoS One. 2018 Feb 28;13(2):e0193478. doi: 10.1371/journal.pone.0193478 (PMC5831102; doi:10.1371/journal.pone.0193478)
Supplement: S1 Table — Bivariate correlations reflect Pearson’s r coefficients. Item numbers reflect numbering from ProQOL instrument manual [1]. CS = Compassion Satisfaction. BO = Burnout. STS = Secondary Traumatic Stress. M = sample mean, SD = standard deviation. * p < .05. ** p < .01. (DOCX) [file pone.0193478.s001.docx]

| Item | 3CS | 6CS | 12CS | 16CS | 18CS | 20CS | 22CS | 24CS | 27CS | 30CS | 1BO | 4BO | 8BO | 10BO | 15BO | 17BO | 19BO | 21BO | 26BO | 29BO | 2STS | 5STS | 7STS | 9STS | 11STS | 13STS | 14STS | 23STS | 25STS | 28STS |
| --- | --- | --- | --- | --- | --- | --- | --- | --- | --- | --- | --- | --- | --- | --- | --- | --- | --- | --- | --- | --- | --- | --- | --- | --- | --- | --- | --- | --- | --- | --- |
| 6CS | .519^**^ |  |  |  |  |  |  |  |  |  |  |  |  |  |  |  |  |  |  |  |  |  |  |  |  |  |  |  |  |  |
| 12CS | .524^**^ | .422^**^ |  |  |  |  |  |  |  |  |  |  |  |  |  |  |  |  |  |  |  |  |  |  |  |  |  |  |  |  |
| 16CS | .426^**^ | .414^**^ | .468^**^ |  |  |  |  |  |  |  |  |  |  |  |  |  |  |  |  |  |  |  |  |  |  |  |  |  |  |  |
| 18CS | .456^**^ | .401^**^ | .469^**^ | .470^**^ |  |  |  |  |  |  |  |  |  |  |  |  |  |  |  |  |  |  |  |  |  |  |  |  |  |  |
| 20CS | .530^**^ | .477^**^ | .463^**^ | .490^**^ | .520^**^ |  |  |  |  |  |  |  |  |  |  |  |  |  |  |  |  |  |  |  |  |  |  |  |  |  |
| 22CS | .528^**^ | .429^**^ | .477^**^ | .491^**^ | .547^**^ | .561^**^ |  |  |  |  |  |  |  |  |  |  |  |  |  |  |  |  |  |  |  |  |  |  |  |  |
| 24CS | .561^**^ | .455^**^ | .489^**^ | .495^**^ | .528^**^ | .573^**^ | .646^**^ |  |  |  |  |  |  |  |  |  |  |  |  |  |  |  |  |  |  |  |  |  |  |  |
| 27CS | .381^**^ | .408^**^ | .365^**^ | .433^**^ | .427^**^ | .430^**^ | .478^**^ | .488^**^ |  |  |  |  |  |  |  |  |  |  |  |  |  |  |  |  |  |  |  |  |  |  |
| 30CS | .528^**^ | .419^**^ | .553^**^ | .500^**^ | .621^**^ | .535^**^ | .537^**^ | .571^**^ | .391^**^ |  |  |  |  |  |  |  |  |  |  |  |  |  |  |  |  |  |  |  |  |  |
| 1BO | .484^**^ | .407^**^ | .408^**^ | .403^**^ | .475^**^ | .446^**^ | .406^**^ | .439^**^ | .329^**^ | .424^**^ |  |  |  |  |  |  |  |  |  |  |  |  |  |  |  |  |  |  |  |  |
| 4BO | .578^**^ | .487^**^ | .406^**^ | .453^**^ | .458^**^ | .458^**^ | .458^**^ | .481^**^ | .398^**^ | .441^**^ | .551^**^ |  |  |  |  |  |  |  |  |  |  |  |  |  |  |  |  |  |  |  |
| 8BO | -.125^**^ | -.069^*^ | -.121^**^ | -.167^**^ | -.176^**^ | -.124^**^ | -.149^**^ | -.137^**^ | -.091^**^ | -.159^**^ | -.357^**^ | -.169^**^ |  |  |  |  |  |  |  |  |  |  |  |  |  |  |  |  |  |  |
| 10BO | -.336^**^ | -.251^**^ | -.358^**^ | -.303^**^ | -.403^**^ | -.295^**^ | -.310^**^ | -.338^**^ | -.201^**^ | -.448^**^ | -.445^**^ | -.295^**^ | .474^**^ |  |  |  |  |  |  |  |  |  |  |  |  |  |  |  |  |  |
| 15BO | .272^**^ | .290^**^ | .306^**^ | .385^**^ | .243^**^ | .304^**^ | .280^**^ | .296^**^ | .287^**^ | .286^**^ | .235^**^ | .255^**^ | -.028 | -.080^**^ |  |  |  |  |  |  |  |  |  |  |  |  |  |  |  |  |
| 17BO | .411^**^ | .373^**^ | .390^**^ | .511^**^ | .578^**^ | .501^**^ | .437^**^ | .470^**^ | .445^**^ | .495^**^ | .552^**^ | .492^**^ | -.231^**^ | -.387^**^ | .297^**^ |  |  |  |  |  |  |  |  |  |  |  |  |  |  |  |
| 19BO | -.162^**^ | -.165^**^ | -.183^**^ | -.241^**^ | -.276^**^ | -.247^**^ | -.195^**^ | -.166^**^ | -.105^**^ | -.297^**^ | -.336^**^ | -.191^**^ | .329^**^ | .471^**^ | -.045 | -.299^**^ |  |  |  |  |  |  |  |  |  |  |  |  |  |  |
| 21BO | -.153^**^ | -.178^**^ | -.197^**^ | -.237^**^ | -.278^**^ | -.210^**^ | -.195^**^ | -.159^**^ | -.106^**^ | -.233^**^ | -.354^**^ | -.200^**^ | .346^**^ | .395^**^ | -.064^*^ | -.266^**^ | .599^**^ |  |  |  |  |  |  |  |  |  |  |  |  |  |
| 26BO | -.178^**^ | -.151^**^ | -.196^**^ | -.260^**^ | -.412^**^ | -.252^**^ | -.236^**^ | -.228^**^ | -.095^**^ | -.339^**^ | -.358^**^ | -.250^**^ | .290^**^ | .455^**^ | -.028 | -.356^**^ | .507^**^ | .549^**^ |  |  |  |  |  |  |  |  |  |  |  |  |
| 29BO | .497^**^ | .340^**^ | .390^**^ | .366^**^ | .313^**^ | .421^**^ | .421^**^ | .488^**^ | .325^**^ | .512^**^ | .315^**^ | .400^**^ | -.043 | -.153^**^ | .273^**^ | .350^**^ | -.060^*^ | -.058^*^ | -.122^**^ |  |  |  |  |  |  |  |  |  |  |  |
| 2STS | .148^**^ | .080^**^ | .064^*^ | .072^*^ | .045 | .105^**^ | .078^**^ | .115^**^ | .057^*^ | .101^**^ | .032 | .130^**^ | .202^**^ | .155^**^ | .053 | .069^*^ | .209^**^ | .214^**^ | .087^**^ | .120^**^ |  |  |  |  |  |  |  |  |  |  |
| 5STS | .031 | .056^*^ | -.017 | -.059^*^ | -.067^*^ | -.074^**^ | -.033 | -.008 | -.004 | -.044 | -.132^**^ | -.007 | .287^**^ | .260^**^ | .046 | -.128^**^ | .265^**^ | .185^**^ | .148^**^ | .032 | .223^**^ |  |  |  |  |  |  |  |  |  |
| 7STS | -.011 | .051 | -.076^**^ | -.090^**^ | -.089^**^ | -.038 | -.049 | -.061^*^ | .014 | -.068^*^ | -.210^**^ | -.044 | .463^**^ | .399^**^ | .034 | -.137^**^ | .332^**^ | .295^**^ | .228^**^ | .004 | .324^**^ | .276^**^ |  |  |  |  |  |  |  |  |
| 9STS | -.101^**^ | -.059^*^ | -.109^**^ | -.190^**^ | -.171^**^ | -.143^**^ | -.118^**^ | -.107^**^ | -.042 | -.166^**^ | -.316^**^ | -.141^**^ | .579^**^ | .541^**^ | .023 | -.280^**^ | .427^**^ | .391^**^ | .376^**^ | -.049 | .244^**^ | .290^**^ | .466^**^ |  |  |  |  |  |  |  |
| 11STS | -.200^**^ | -.148^**^ | -.236^**^ | -.228^**^ | -.274^**^ | -.204^**^ | -.220^**^ | -.244^**^ | -.116^**^ | -.279^**^ | -.405^**^ | -.214^**^ | .527^**^ | .697^**^ | -.032 | -.309^**^ | .468^**^ | .423^**^ | .442^**^ | -.075^**^ | .195^**^ | .283^**^ | .473^**^ | .631^**^ |  |  |  |  |  |  |
| 13STS | -.108^**^ | -.089^**^ | -.104^**^ | -.186^**^ | -.202^**^ | -.157^**^ | -.171^**^ | -.162^**^ | -.124^**^ | -.187^**^ | -.329^**^ | -.196^**^ | .521^**^ | .492^**^ | -.020 | -.278^**^ | .398^**^ | .379^**^ | .315^**^ | -.044 | .186^**^ | .260^**^ | .418^**^ | .600^**^ | .586^**^ |  |  |  |  |  |
| 14STS | -.101^**^ | -.081^**^ | -.118^**^ | -.154^**^ | -.168^**^ | -.106^**^ | -.112^**^ | -.120^**^ | -.072^*^ | -.162^**^ | -.305^**^ | -.159^**^ | .532^**^ | .460^**^ | -.008 | -.209^**^ | .358^**^ | .336^**^ | .271^**^ | -.033 | .204^**^ | .259^**^ | .382^**^ | .541^**^ | .501^**^ | .702^**^ |  |  |  |  |
| 23STS | -.136^**^ | -.077^**^ | -.200^**^ | -.147^**^ | -.185^**^ | -.140^**^ | -.125^**^ | -.162^**^ | -.089^**^ | -.159^**^ | -.296^**^ | -.169^**^ | .429^**^ | .397^**^ | -.038 | -.216^**^ | .306^**^ | .290^**^ | .250^**^ | -.093^**^ | .156^**^ | .244^**^ | .311^**^ | .427^**^ | .427^**^ | .460^**^ | .485^**^ |  |  |  |
| 25STS | -.197^**^ | -.174^**^ | -.187^**^ | -.241^**^ | -.223^**^ | -.193^**^ | -.178^**^ | -.213^**^ | -.109^**^ | -.225^**^ | -.392^**^ | -.234^**^ | .478^**^ | .507^**^ | -.102^**^ | -.276^**^ | .363^**^ | .346^**^ | .360^**^ | -.094^**^ | .136^**^ | .239^**^ | .322^**^ | .482^**^ | .566^**^ | .525^**^ | .526^**^ | .563^**^ |  |  |
| 28STS | -.080^**^ | -.090^**^ | -.071^*^ | -.109^**^ | -.128^**^ | -.115^**^ | -.093^**^ | -.094^**^ | -.014 | -.120^**^ | -.168^**^ | -.130^**^ | .300^**^ | .305^**^ | .012 | -.166^**^ | .211^**^ | .201^**^ | .221^**^ | -.025 | .110^**^ | .170^**^ | .229^**^ | .306^**^ | .315^**^ | .345^**^ | .365^**^ | .311^**^ | .381^**^ |  |
| *M* | 4.363 | 3.709 | 4.097 | 3.820 | 3.744 | 3.790 | 3.914 | 4.190 | 3.518 | 4.134 | 4.156 | 3.999 | 1.692 | 1.813 | 3.574 | 3.692 | 2.912 | 2.880 | 2.660 | 4.350 | 3.190 | 2.536 | 2.233 | 2.147 | 1.823 | 1.787 | 1.603 | 1.635 | 1.386 | 1.765 |
| *SD* | 0.678 | 0.945 | 0.909 | 0.891 | 0.909 | 0.848 | 0.904 | 0.830 | 0.996 | 0.929 | 0.770 | 0.836 | 0.891 | 1.001 | 1.282 | 0.915 | 1.104 | 1.131 | 1.248 | 0.723 | 1.103 | 1.061 | 1.030 | 0.952 | 0.950 | 0.874 | 0.815 | 0.879 | 0.732 | 0.911 |
