# Supplementary material for: The ProQOL-21: A revised version of the Professional Quality of Life (ProQOL) scale based on Rasch analysis
Source: PLoS One. 2018 Feb 28;13(2):e0193478. doi: 10.1371/journal.pone.0193478 (PMC5831102; doi:10.1371/journal.pone.0193478)
Supplement: S1 Results — This set of supplementary results details the attempts at examining whether the Burnout and Secondary Traumatic Stress measures in the ProQOL conformed to the assumptions of a Rasch model. (DOCX) [file pone.0193478.s002.docx]

**Supplementary Results**

**Burnout**

In the initial model, person-reliability (.74-.80) and separation (1.68-1.97) suggested average model fit, with adequate Cronbach alpha reliability (*a* = .81). The measure targeting (-1.02, Model *SE* = .42) suggested that, on average, nurses found the burnout items difficult to endorse (i.e., provide responses more-indicative of burnout). Several potential avenues for measure improvement were noted at this stage. The empirical item-category chart and category structure estimates suggested potential poor fit and coherence of the ‘Very Often’ (5% of responses) and ‘Often’ (8% of responses) options for many of the items. Furthermore, individual items seemed to suggest overfit or underfit with the latent factor (outfit > 1.40; infit < 0.60), and were therefore potential candidates for deletion. Lastly, the univariate dimensionality of the burnout factor was questionable, as the first contrast of residuals stemming from a principle components analysis suggested a noteworthy second factor (Eigenvalue = 2.53). In the interest of retaining as many relevant items as possible to potentially better target the range of participant responses on the burnout factor, we decided to address the response category issues first, before moving on to consider item deletion or splitting the burnout scale according to the residual factor structure. Category restructuring produced minor improvements in person reliability (.76-.81), separation (1.80-2.05), and Cronbach alpha reliability (*a* = .82), and similarly improved the measure targeting (-.59, Model *SE* = .53). Despite item and measure improvements following the category restructuring, the unidimensionality of the burnout measure was still questionable (Eigenvalue = 2.49). Looking at the pattern of loadings on the first non-marginal contrast, an interpretable pattern of variables that evenly loaded on two factors emerged. One factor, represented most strongly by the items “I feel worn out because of my work as a (helper)” and “I feel overwhelmed because my case (work) load seems endless” seemed to focus on the work-referential facets of burnout. Contrastingly the other factor was represented by items such as “I (do not) feel connected to others” and “I am (not) a very caring person”, which seemed to suggest self-referential facets of burnout. We therefore split the items of the burnout scale into these new subsets and proceeded with further data analysis. No further dimensionality issues were noted for the remainder of the analyses, and no evidence of response dependency was observed among items in each of the new subsets.

**Self-Referential Burnout.** The first subset of items reflecting self-referential facets of burnout (consisting of item 1, 4, 15, 17, and 29) initially demonstrated less-good model fit. The person-reliability was poor (.65-.72) and demonstrate mediocre separation (1.35-1.61), with Cronbach’s alpha similarly confirming the less-than-optimal reliability (*a* = .76). Coefficients of item misfit suggested that Item 15, “I (do not) have beliefs that sustain me” was a particularly poorly-loading item on the self-referential burnout scale (mean square infit = 1.29; outfit = 1.52). As over 50% of the variance in this item seemed to be targeting factors outside of self-referential burnout, we removed this item, which provided a small enhancement to model fit (person-reliability = .65-.73; separation = 1.35-1.64; Cronbach’s alpha *a* = .78). Item reliability (0.99) and separation (8.30-8.61) did not suggest problems of fit. No other items indicated noteworthy misfit (.60 < X < 1.40; [1]), suggesting that the small item pool was likely a key contributor to the mediocre model fit. The targeting of the measure (-0.86, Model *SE* = .999) suggested that nurses found these items difficult to endorse, and that the indicators of lower-levels of self-referential burnout were not well-represented in the item content of the reduced pool of four items. In summary, model and item adequacy were optimised where possible, and while most assumptions of the Rasch model were met at this stage, the mediocre reliability outlined prior was likely an indicator of the limited item pool.

In a manner consistent with the compassion satisfaction scale, tests of DIF for the four self-referential burnout items were conducted on the basis of nurse gender, age category (<mean; <50 years), and nurse grade. Comparisons between nurses on the basis of these categories indicated either no or neglible indication of DIF (2, 3), as the DIF contrast in each instance was <0.43 logits. Therefore no evidence of item bias on the basis of participant demographic characteristics was noted.

**Work-Referential Burnout**. The second subset of burnout items that reflected work-referential burnout (consisting of items 8, 10, 19, 21, and 26) demonstrated mediocre model fit, although not to the same degree as the self-referential burnout subset of items. Person-reliability (.72-.78) and separation (1.61-1.91) coefficients suggested mediocre reliability, with Cronbach’s alpha (*a* = .80) suggesting adequate reliability. The work-referential burnout items were better targeted (-0.44, Model *SE* = .70) in comparison to the self-referential items, which suggested that nurses found these items indicative of the nurses’ average work-referential burnout. No items indicated misfit on the basis of infit and outfit mean-square coefficient suitability for a Likert-style measure. An initial inspection of the response categories suggested that several items would benefit from collapsing of one or more response categories. Items 8, 10, 21, and 26 functioned closer to the recommendations for well-functioning response categories by Linacre [4] by collapsing the ‘Often’ and ‘Very Often’ response categories. All modified response categories were ordered in difficulty, fulfilling the assumption of monotonically ordered categories [1]. No evidence of multidimensionality was noted (Eigenvalues < 2.0), and no evidence of item dependency was found on the basis of small standardised residual correlations between items. In sum, these findings suggested that the limited item pool of the work-referential burnout subscale did not impede the measurement properties notably, although the person-level reliability of the measure was mediocre.

Tests of DIF were conducted using the same demographic categories for nurses outlined in the self-referential burnout scale prior. Tests of DIF on the basis of nurse grade, and nurse age (both mean and <50 years categorisations), and nurse gender did not suggest DIF for any of the items beyond that of a negligible quantity per the severity categorisations of Zwick et al. (DIF contrast ≥ 0.43 logits; [3]).

**Secondary Traumatic Stress**

The initial examination of the secondary traumatic stress (STS) scale suggested that nurses found the items difficult to endorse, due to the negative person-level targeting coefficient (-1.47, Model *SE* = .49). Indicators of person-reliability (.77-.82) and person-separation (1.82-2.13) suggested average-to-good reliability of the person estimates, with Cronbach alpha (*a* = .85) further confirming the overall model of STS. Consistent with the previous analyses, item-reliability (1.00) and item-separation (19.29-20.30) were all excellent. We confirmed an underlying univariate factor structure, and the absence of item dependency, for the STS scale items. However, several misfitting items were identified within the scale, and appeared to fail the Rasch model specifications for validity.

Item misfit analysis suggested that the STS scale had several items that were capturing variance beyond that of the intended underlying STS factor (i.e., outfit > 1.40), or demonstrated noteworthy differences between person estimates of ability and their actual scores on the items (i.e., infit > 1.40). The first of these potentially misfitting items was item 2, “I am preoccupied with more than one person I (help)”, which appeared to be a valid candidate for deletion on the basis of its infit and outfit mean square values (infit_mnsq_ = 1.55; outfit_mnsq_ = 1.55). Removing this item led to a minor decrease in person-level reliability (.75-.81), separation (1.75-2.08), and Cronbach alpha (*a* = .85). A second iteration of examining item misfit flagged item 5 “I jump or am startled by unexpected sounds” (infit_mnsq_ = 1.70; outfit_mnsq_ = 1.70) and item 28 “I can't recall important parts of my work with trauma victims” (infit_mnsq_ = 1.52; outfit_mnsq_ = 1.66) as potential candidates for deletion due to large infit and outfit mean square coefficients (>1.40). Removing both items from the STS scale had a minor impact on person-level reliability (.75-.80), separation (1.71-2.02), and Cronbach alpha (*a* = .87). A third and final iteration of examining item misfit suggested item 7 “I find it difficult to separate my personal life from my life as a (helper)” (infit_mnsq_ = 1.46; outfit_mnsq_ = 1.47) and item 23 “I avoid certain activities or situations because they remind me of frightening experiences of the people I (help)” (infit_mnsq_ = 1.32; outfit_mnsq_ = 1.51) demonstrated similar issues with poor fit as indicated by large mean square coefficients. Removal of these items led to a further, albeit minor, decrement in person-level reliability (.72-.79), separation (1.59-1.94), with no change in Cronbach alpha (*a* = .87). Item-level reliability (0.99) and separation (13.44-13.62) remained good indicators of model fit. The removal of several ‘easier’ indicators of STS (i.e., nurses found it easier to provide positive responses to these items) led to the targeting of the scale falling in a notably more negative direction, (-2.56, Model SE = .87). We therefore decided to assess the prospect of improving measure targeting on the now-reduced item subset (items 9, 11, 13, 14, and 25) reflective of secondary traumatic stress

We examined the category response functions for each item against the well-functioning response category guidelines of Linacre [4]. Item 9 did not benefit from any category collapsing, and generally demonstrated adequate response category functioning. Items 11, 13, and 14 demonstrated fragility with the ‘Often’ and ‘Very Often’ responses at the positive of the response scale, and collapsing these response options led to improvements in category fit and coherence coefficients. After systematically testing a similar collapsing of the response options of the positive end of the scale, Item 25’s category response functioning appeared to benefit from collapsing all categories beyond the ‘Never’ response into one inclusive category, converting the item into a dichotomous response item (‘Never’ and ‘Not Never’). The final set of modified response categories for the five items demonstrated improved measure targeting (-1.60, Model *SE* = .92). The person-reliability (.73-.80), separation (1.64-1.98), and Cronbach reliability (*a* = .86) demonstrated generally minor improvements as a consequence of the modified response categories. Item-level reliabilities (.98-.98) and separation (6.27-6.35) suggested that the refined measure demonstrated good fit despite the reduced item response categories. We therefore decided to continue with the final tests of differential item functioning on the reduced item subset (items 9, 11, 13, 14, and 25) reflective of secondary traumatic stress.

Examination of DIF for the reduced STS item pool indicated no differential responding based on nurse age (mean or <50 years categorisations), or nurse grade. We found evidence of gender-based DIF for items 9, 11, and 25 on the basis of significant Mantel chi-square and Rasch-Welch probabilities (*p* < .05). Item 25 (“As a result of my (helping), I have intrusive, frightening thoughts”) presented the most-severe demonstration of DIF (DIF contrast = 0.63 logits), reflecting moderate to large differential responding [3]. Female nurses reported much higher scores on this item in comparison to their male counterparts, therefore we split the item into two to model the item’s differential responding [5]. The revised model alleviated DIF concerns with item 9 (DIF contrast = 0.39 logits, or negligible DIF; [3]), however item 11 remained indicative of differential responding due to gender (DIF contrast = 0.56 logits, or slight to moderate DIF). Item 11 (“Because of my (helping), I have felt "on edge" about various things”) again favoured higher-than-anticipated scores for female nurses compared to male nurses, and was split on the basis of participant gender before being re-examined. The final model did not demonstrate any further evidence of DIF on account of participant gender. Based on the generally limited pool of items (five or four per measure) that indicated adequate fit, with less-adequate person-reliability and targeting in each instance, we re-evaluated the analysis of the ProQOL as described in our Results.

**References**

1. Bond TG, Fox CM. Applying the Rasch model: Fundamental measurement in the human sciences. 3rd ed. New York, NY: Routledge; 2015.

2. Linacre JM. A user's guide to Winsteps Ministep Rasch-model computer programs 2016. Available from: http://www.winsteps.com.

3. Zwick R, Thayer DT, Lewis C. An Empirical Bayes Approach to Mantel-Haenszel DIF Analysis. Journal of Educational Measurement. 1999;36(1):1-28.

4. Linacre JM. Optimising rating scale category effectiveness. Journal of Applied Measurement. 2002;3(1):85-106.

5. Hagquist C, editor Psychometric properties of the PsychoSomatic Problems scale–an examination using the Rasch model. The ACSPRI Social Science Methodology Conference 2006 The University of Sydney, Sydney, Australia 10-13 December 2006; 2006.
